# Incidence of Hospitalized Pneumococcal Pneumonia among Adults in Guatemala, 2008-2012

**DOI:** 10.1371/journal.pone.0140939

**Published:** 2015-10-21

**Authors:** Carmen Lucía Contreras, Jennifer R. Verani, María Renee Lopez, Antonio Paredes, Chris Bernart, Fabiola Moscoso, Aleida Roldan, Wences Arvelo, Kim A. Lindblade, John P. McCracken

**Affiliations:** 1 Center for Health Studies, Universidad del Valle de Guatemala, Guatemala City, Guatemala; 2 National Center for Immunization and Respiratory Diseases, Centers for Disease Control and Prevention (CDC), Atlanta, GA, United States of America; 3 National Center for Epidemiology, Ministry of Public Health and Social Welfare (MSPAS), Guatemala City, Guatemala; 4 Division of Global Disease Detection and Emergency Response, Centers for Disease Control and Prevention (CDC), Atlanta GA, United States of America; University of Cambridge, UNITED KINGDOM

## Abstract

**Background:**

*Streptococcus pneumoniae* is a leading cause of pneumonia worldwide. However, the burden of pneumococcal pneumonia among adults in low- and middle-income countries is not well described.

**Methods:**

Data from 2008–2012 was analyzed from two surveillance sites in Guatemala to describe the incidence of pneumococcal pneumonia in adults. A case of hospitalized pneumococcal pneumonia was defined as a positive pneumococcal urinary antigen test or blood culture in persons aged ≥ 18 years hospitalized with an acute respiratory infection (ARI).

**Results:**

Among 1595 adults admitted with ARI, 1363 (82%) had either urine testing (n = 1286) or blood culture (n = 338) performed. Of these, 188 (14%) had pneumococcal pneumonia, including 173 detected by urine only, 8 by blood culture only, and 7 by both methods. Incidence rates increased with age, with the lowest rate among 18–24 year-olds (2.75/100,000) and the highest among ≥65 year-olds (31.3/100,000). The adjusted incidence of hospitalized pneumococcal pneumonia was 18.6/100,000 overall, with in-hospital mortality of 5%.

**Conclusions:**

An important burden of hospitalized pneumococcal pneumonia in adults was described, particularly for the elderly. However, even adjusted rates likely underestimate the true burden of pneumococcal pneumonia in the community. These data provide a baseline against which to measure the indirect effects of the 2013 introduction of the pneumococcal conjugate vaccine in children in Guatemala.

## Introduction

Pneumonia is a leading cause of death worldwide, and *Streptococcus pneumoniae* is a primary etiology [1–3]. The global burden of pneumococcal pneumonia among young children has been well characterized, with the highest incidence of disease and death occurring in low- and middle- income countries [4]. Sparse data are available for adults in developing countries, despite older adults and those with chronic illness being at high risk for pneumococcal disease. Estimates of the proportion of hospitalized adult pneumonia caused by pneumococcus have ranged from 17% to 45% [5, 6]. However, determining the etiology of pneumonia is challenging since diagnostic tools have important limitations [7–9], and the most sensitive of these tools are not typically available in resource-poor settings.

Evidence from high-income countries shows that vaccinating infants with pneumococcal conjugate vaccine (PCV) can prevent pneumococcal disease in adults through herd protection since vaccinated children are less likely to be colonized with and transmit *S*. *pneumoniae* [10–12]. However, it is unknown whether similar indirect protection will occur in low- and middle-income countries, given the greater force of transmission, poor underlying health status and low vaccine coverage. In Guatemala, a 13-valent PCV (PCV13) was introduced in November 2012 for children ≤1 year old. As a lower-middle income country not eligible for support from the Global Alliance for Vaccination and Immunization [13], it is important to demonstrate the impact of PCV13 introduction in this setting, including direct and indirect effects, in order to justify investment in the vaccine and guide decisions about sustained use. This study describes the incidence of hospitalized pneumococcal pneumonia in adults to provide insight into the pre-PCV burden.

## Materials and Methods

### Study area and design

The International Emerging Infections Program, a collaboration between the Universidad del Valle de Guatemala (Guatemala City, Guatemala), the United States Centers for Disease Control and Prevention (Atlanta, GA) and the Guatemalan Ministry of Public Health and Welfare (Guatemala City, Guatemala), conducts active, hospitalized-based surveillance for acute respiratory infections (ARI) in two sites in Guatemala, as has been described previously [14]. Briefly, surveillance in the Department of Santa Rosa started in November 2007 and is conducted at the only hospital in the department, the National Hospital of Cuilapa (elevation approximately 900 m). In Quetzaltenango, surveillance for hospitalized ARI began in February 2009 and is conducted at the Western Regional Hospital (elevation approximately 2300 m), one of two public hospitals in the department. Both surveillance hospitals provide free healthcare and serve mostly low- and mid-income populations.

At the hospitals, trained surveillance nurses search daily in logbooks in the emergency rooms and inpatient wards to identify patients with respiratory disease. Patients admitted to surveillance hospitals with evidence of acute infection (e.g. fever, elevated white blood cell count) and at least one sign or symptom of respiratory disease (e.g., cough or difficulty breathing) were considered ARI cases (Table 1). Enrolled patients were interviewed about demographic, risk factor and health history information. Additional data were abstracted from the medical record. A study physician performed a respiratory physical examination on all patients who met the case definition. When feasible, study nurses measured peripheral oxygen saturation using a pulse oximeter with the patient off oxygen. Urine samples were collected from ARI patients aged ≥ 18 and tested using BinaxNOW^®^ (Binax, Inc., Portland, Maine), a rapid immunochromatographic test (ICT) that detects *S*. *pneumoniae* C polysaccharide antigen. Nasopharyngeal and oropharyngeal (NP/OP) swabs were also collected and tested using a real-time probe-hydrolysis (TaqMan^®^) real-time reverse transcription PCR (rRT-PCR) assay to detect eight respiratory viruses (syncytial virus (RSV), human metapneumovirus, adenovirus, human parainfluenza virus 1–3, influenza virus A and B). In addition, at the discretion of the treating physician, patients may have had a blood culture and/or chest x-ray performed. For study participants, chest x-rays were interpreted by a panel of Guatemalan radiologists using an adaptation of World Health Organization guidelines for standardized interpretation of pediatric chest X-rays in order to identify likely bacterial pneumonia [15].

**Table 1:** Case definition for acute respiratory infection (ARI)*, Guatemala, 2008–2012.

Signs of acute infection	Signs or symptoms of respiratory disease
Fever (≥38°C)	Signs
Hypothermia (<35.5°C)	Abnormal breath sounds
Abnormal white blood cell (WBC) count < 3000 or >11000/mm3	Tachypnea (≥20/minute)
Abnormal WBC differential	Symptoms
	Cough
	Sputum production
	Pleuritic chest pain
	Hemoptysis
	Difficulty breathing
	Shortness of breath
	Sore throat

*ARI case definition: hospitalized patient with at least one sign of acute infection and at least one sign or symptom of respiratory disease.

### Analysis

The analysis included enrolled ARI cases, aged ≥18 years with either ICT or blood culture results available. A case of pneumococcal pneumonia was defined as an ARI case with either a positive ICT or a blood culture that grew *S*. *pneumoniae*. Data from January 2008 for Santa Rosa and from February 2009 for Quetzaltenango, through December 2012 for both study sites, were included in this analysis. Characteristics and outcomes of patients with pneumococcal pneumonia were described.

The incidence rate of hospitalized pneumococcal pneumonia was estimated by year, age group and study site. Denominators were the age-specific total populations of the municipalities in the surveillance catchment area obtained from the 2002 national census adjusted for population growth, accounting for an 11-month period of surveillance in Quetzaltenango in 2009 (data in S1 Dataset). Incidence estimates were restricted to cases residing in a surveillance catchment area for which a healthcare utilization survey had been carried out when surveillance was initiated at each site. The surveys found that among persons aged ≥ 5 years hospitalized with pneumonia or severe respiratory disease during the prior 12 months, 75% in Santa Rosa and 50% in Quetzaltenango reported being admitted to the surveillance hospital [14, 16, 17]. In order to adjust estimates of hospitalized pneumococcal pneumonia incidence for those cases seeking care elsewhere, observed case counts were divided by 0.75 and 0.50 in Santa Rosa and Quetzaltenango, respectively. The incidence was further adjusted to account for missing test results and proportion of eligible patients enrolled, and these adjustments were made by year. Case-patients residing outside the catchment area were included in the descriptive analysis but were not included in incidence calculations. All analyses were performed in R (version 2.13.1).

### Ethics

The protocol was approved by the institutional review boards of the Universidad del Valle de Guatemala and the Centers for Disease Control and Prevention, and reviewed by the Guatemala Ministry of Public Health. Verbal consent was requested of patients in order to screen them for eligibility. Written, informed consent was obtained from eligible patients willing to participate.

## Results

**Fig 1:**
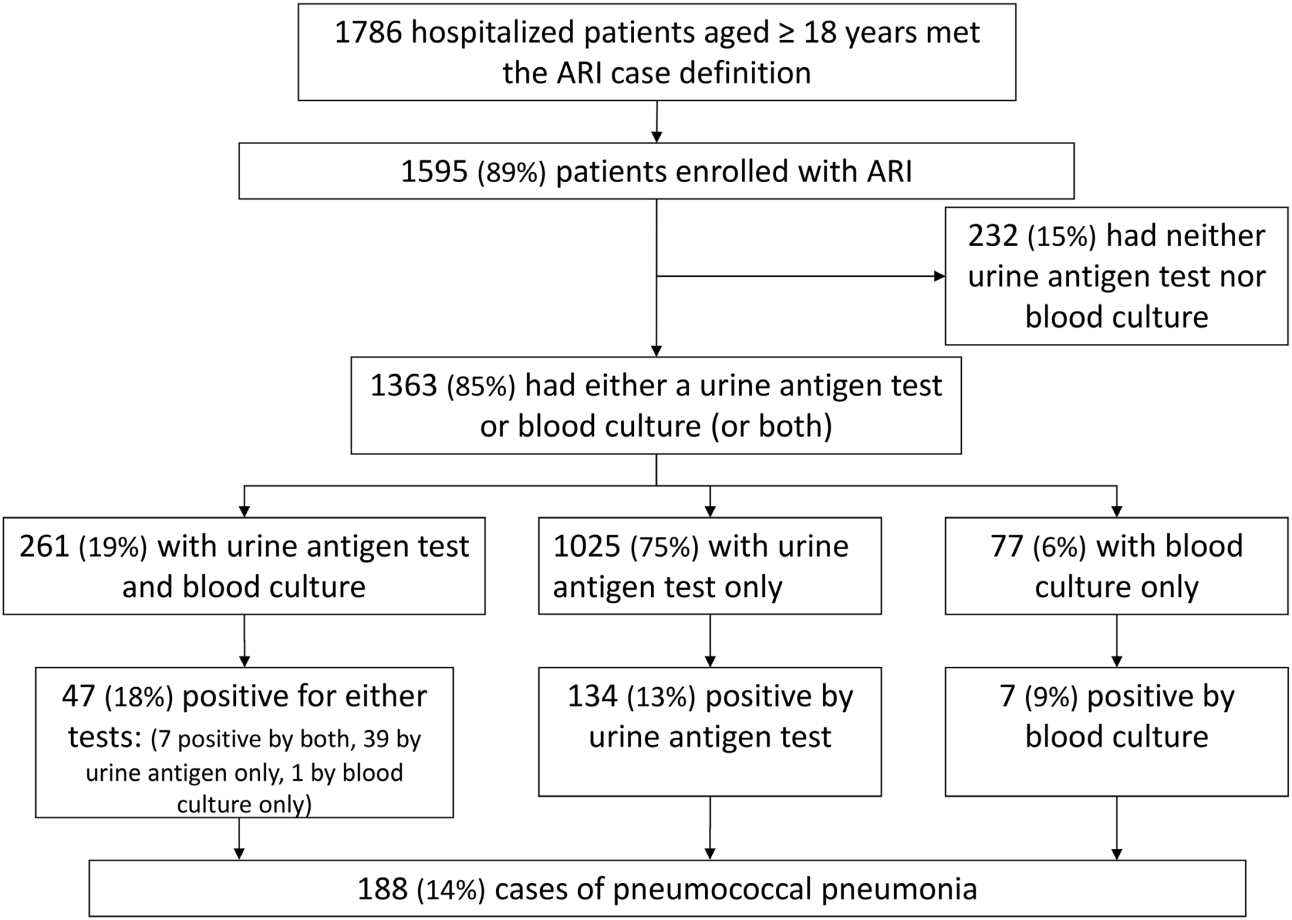
Ascertainment of pneumococcal pneumonia within hospital surveillance of acute respiratory infection (ARI), Guatemala, 2008–2012. Flow diagram of patients included in this analysis.

From January 1, 2008 through December 31, 2012, a total of 1786 hospitalized patients aged ≥18 years met the ARI case definition for possible inclusion at the two hospitals, and 1595 (89%) were enrolled (Fig 1). Among 1363 case-patients with blood culture or urine antigen testing, 1025 (75%) were tested by urine antigen test only, 77 (6%) by blood culture only, and 261 (19%) by both methods. A total of 188 (14%) cases of laboratory-confirmed pneumococcal pneumonia were detected.

More than one-third of pneumococcal pneumonia cases (n = 71, 38%) were aged ≥ 65 years, and slightly less than half (n = 88, 47%) were male (Table 2) (data in S2 Dataset). The majority of patients (84%) resided in households where the average monthly income was less than 1,000 Quetzals, (USD ~$130). Cough, reported in 92% of cases, was the most common symptom, followed by difficulty breathing (84%). Reported fever (72%) was more common than measured fever (43%); of note 29 (50%) of cases reported having taken antipyretics. The most common physical finding was tachypnea (respiratory rate >20 breaths per minute) (64%), and relatively few case-patients (11%) had rales, rhonchi or crackles on exam.

**Table 2:** Characteristics of patients with pneumococcal pneumonia, n = 188.

	n/N (%)
**Demographic characteristics**	
Age (years)	
18 to 39	55/188 (29)
40 to 64	62/188 (33)
≥ 65	71/188 (38)
Male	88/188 (47)
Quetzaltenango	101/188 (54)
Santa Rosa	87/188 (46)
**Risk factors**	
Current smoker	35/186 (19)
Secondhand smoke exposure	28/186 (15)
One or more comorbidities	68/185 (37)
Chronic respiratory disease^	31/184 (17)
Diabetes	21/183 (11)
Chronic cardiovascular disease^^	30/185 (16)
**Socioeconomic status**	
Monthly family income < USD $130	140/166 (84)
Overcrowding (≥ 3 persons per bedroom)	54/186 (29)
Electricity in home	166/186 (89)
Dirt floor	61/186 (33)
Completed primary school	22/186 (12)
Completed high school	10/186 (5)
**Signs, symptoms and physical exam findings**	
Cough	170/185 (92)
Difficulty breathing	155/185 (84)
Reported fever	135/187 (72)
Measured fever ≥ 38°C	80/187 (43)
Tachypnea (≥ 20 breaths/min)	117/182 (64)
Hypoxemia†	71/163 (44)
Rales, crackles or rhonchi on lung exam	20/182 (11)
Wheezing on lung exam	53/182 (29)
**Testing results**	
Abnormal white blood cell count <3000/mm^3^	3/185 (2)
Abnormal white blood cell count >11000/mm^3^	114/185 (62)
Consolidation or large effusion on chest x-rays	80/123 (65)
**Detection of other respiratory viruses**	
Respiratory syncytial virus	8/186 (4)
Human metapneumovirus	7/186 (4)
Parainfluenza virus 1, 2, or 3	13/186 (7)
Adenovirus	11/186 (6)
Influenza virus A or B	20/186 (11)
**Outcome**	
Intensive care unit	19/180 (11)
Mechanical ventilation	15/180 (8)
Hospitalized ≥ 1 week	86/182 (47)
Death (in hospital)	10/183 (5)

^ Includes asthma or lung disease

^^ Includes cardiovascular disease or hypertension

^†^ Oxygen saturation <90% in Santa Rosa and <88% in Quetzaltenango, adjusted for elevation

Consolidation or large effusion on chest x-rays was present in 65% of pneumococcal pneumonia cases (Table 2). A total of 53 (28%) cases had a respiratory virus detected from the NP/OP swab and 7 (13%) had more than one virus present. The frequency of viral pathogens detected was similar in those with no pneumococcal etiology detected (data not shown). Duration of hospitalization ranged from 1 to 80 days (median 7 days). Cases were severe enough to warrant an admission to the intensive care unit for 19 (11%) of patients, and 10 (5%) patients died; the median age of patients who died was 44 years (range 34 to 88 years).

**Fig 2:**
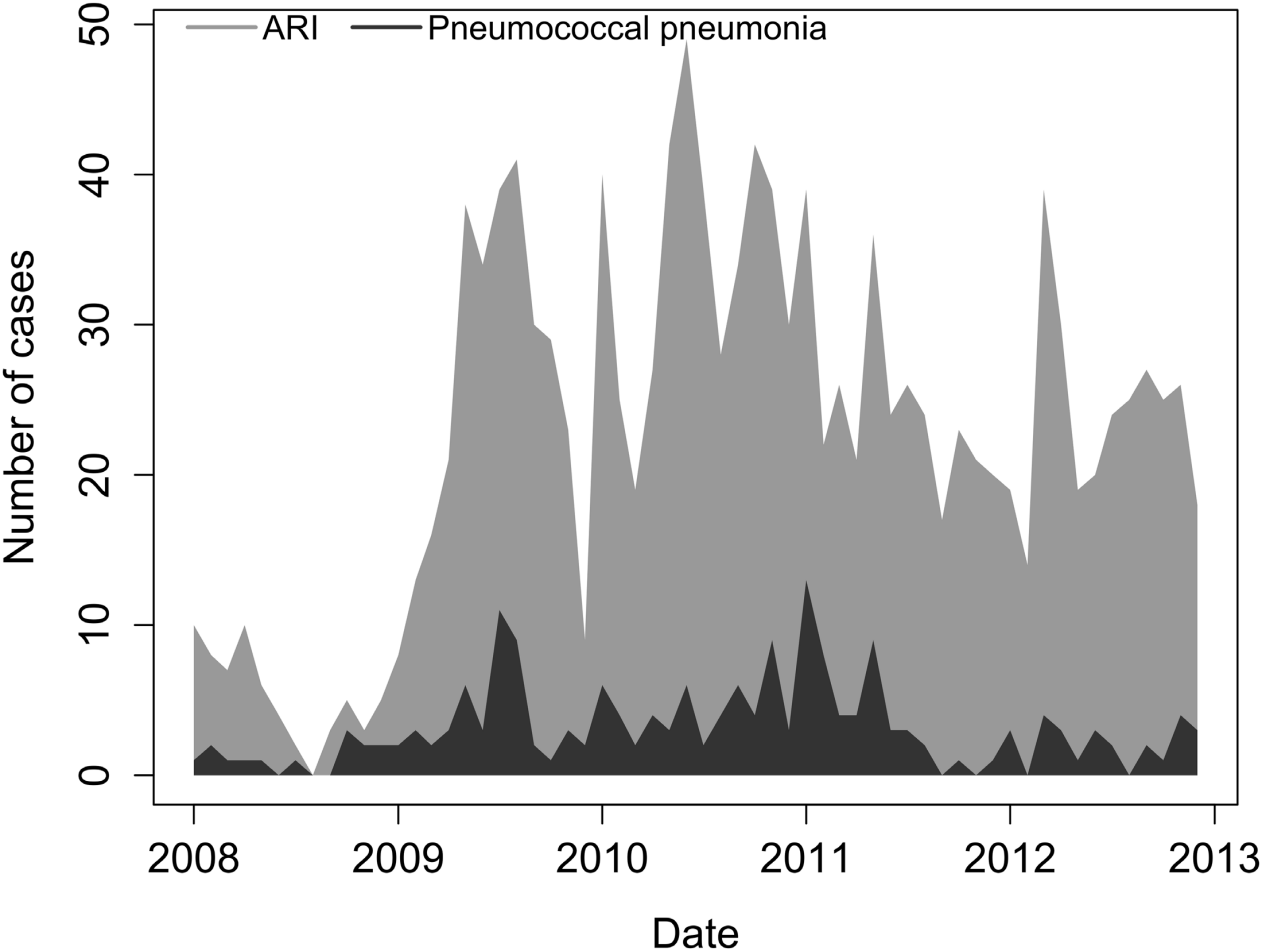
Number of hospitalized ARI tested and pneumococcal pneumonia cases by month, Guatemala, 2008–2012*. Pneumococcal pneumonia cases detected by urine antigen test or blood culture.

Pneumococcal pneumonia cases occurred throughout the year, with no clear seasonal pattern. The timing of peaks of pneumococcal pneumonia cases was similar to that of the peaks in the total number of hospitalized ARI cases over the study period. However, the timing of the peaks varied from year to year (Fig 2) (data in S3 Dataset).

**Fig 3:**
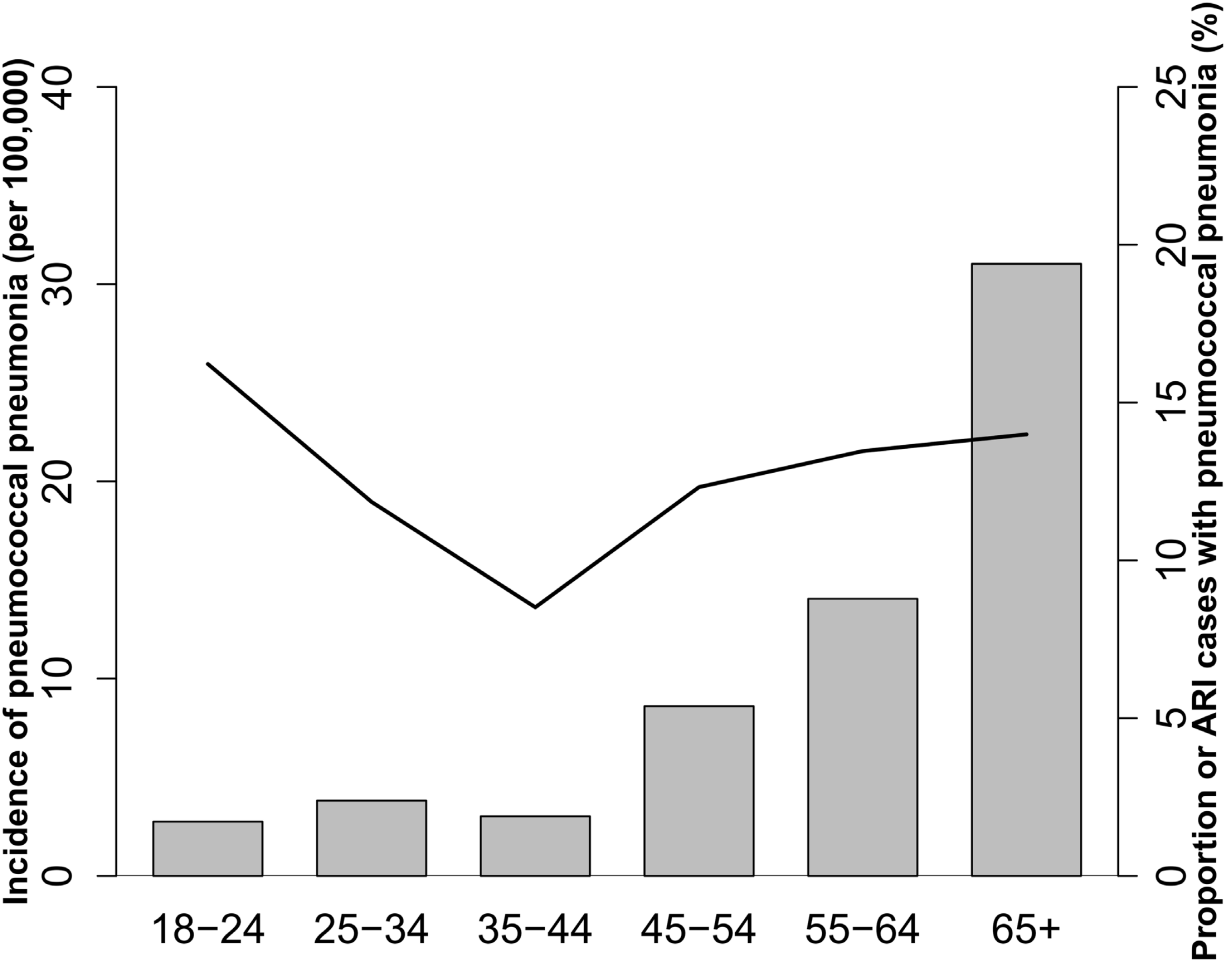
Hospitalized pneumococcal pneumonia incidence rates and proportion of acute respiratory infection (ARI) cases by age, Guatemala, 2008–2012. Incidence rates (bars) ranged from 2.75/100,000 (among 18–24 year-olds) to as high as 31.3 per 100,000 (among adults aged ≥65 years). Proportion of ARI positive for pneumococcus (line) was more stable than the incidence rate across age groups.

Between 2008 and 2012 the observed incidence of hospitalized pneumococcal pneumonia in the defined catchment area ranged from 5.9 to 11.7 cases per 100,000 people (Table 3) (data in S1 and S2 Datasets). Incidence rates adjusted for proportion of population that seeks care at surveillance hospitals in the catchment area, proportion enrolled and tested ranged from 15.3 to 23.1 cases per 100,000 people by site and year. The ranges of annual incidence rates found in Santa Rosa and Quetzaltenango were similar. There was no clear trend in the observed incidence over time (Table 3), with the highest in 2009 in both Santa Rosa (11.7 cases per 100,000) and Quetzaltenango (10.0 cases per 100,000). Correlation was observed in annual incidence rates between the sites, with the ranks of incidence rates by year matching exactly between Santa Rosa and Quetzaltenango during 2009–2012. Incidence rates increased with age, and the highest rate was observed among adults aged ≥65 years (31.3 per 100,000). However, there was no clear trend in the proportion of hospitalized ARI cases with pneumococcal pneumonia by age (Fig 3) (data in S1 Table).

**Table 3:** Observed and adjusted incidence rates of pneumococcal pneumonia cases, Guatemala, 2008–2012.

Department	Year	Cases	Population	Observed incidence per 100,000	Adjusted incidences* per 100,00
Santa Rosa	2008	9	125396	7.2	15.3
	2009	15	128536	11.7	19.1
	2010	13	131928	9.9	17.7
	2011	15	135338	11.1	18.1
	2012	10	138766	7.2	16.7
Quetzaltenango	2009	17	170822	10.0	23.1
	2010	14	192193	7.3	16.9
	2011	17	198202	8.6	22.6
	2012	12	204381	5.9	16.4
Overall		122	1425562	8.6	18.6

* Adjusted for proportions of cases with test results (64%-95%), eligible patients enrolled (81%-98%) and proportion of population that seeks care at surveillance hospitals (50%-75%) (adjustment factors in S2 Table).

## Discussion

This large hospital-based study describes an important burden of hospitalized pneumococcal pneumonia among adults in a middle-income, Latin American country, before PCV introduction in infants. The greatest burden of pneumococcal pneumonia was observed among those ≥65 years (31.3 per 100,000 persons per year), a pattern consistent with other published data [18–22]. Nearly half of patients with pneumococcal pneumonia required hospitalization for a week or more, with an overall mortality of 5%. Of note, the proportion of all ARI cases that tested positive for *S*. *pneumoniae* did not vary widely by age, suggesting that while elderly are at high risk for pneumococcal disease, they are also at high risk for other causes of pneumonia.

Overall in this study 14% of adults hospitalized with an acute respiratory infection had a pneumococcal etiology. A study conducted in Nicaragua that relied primarily upon urine antigen tests to diagnose pneumococcal pneumonia similarly found that 17% of adult community acquired pneumonia was attributable to pneumococcus [23]. Our findings are also consistent with studies from Asia [24] and Africa [25] that have used urine antigen test to diagnose adult pneumococcal pneumonia. However, with a sensitivity of approximately 60–80% and a specificity of 83–97% for pneumococcal pneumonia [3, 9, 26, 27], urine antigen testing likely underestimates the true proportion of pneumonia caused by *S*. *pneumoniae*. Studies relying only on blood culture will greatly underestimate the contribution of pneumococcus [28]. A study from Kenya using latent class analysis, a statistical method for estimating disease burden despite the lack of a gold standard test, estimated that nearly half of adult community acquired pneumonia may be caused by pneumococcus [7]. This suggests that our study underestimates the burden of hospitalized pneumococcal pneumonia even though both blood culture and urine antigen test were performed. Improved diagnostic tools would help establish more accurate and robust estimates of pneumococcal pneumonia burden among adults globally.

Data on the incidence of adult pneumococcal pneumonia from developing countries are extremely limited. A study in two provinces in rural Thailand found an adjusted overall incidence rate of hospitalized pneumococcal pneumonia among persons aged 18 years or older to be 24 per 100,000 person-years [29], which is relatively similar to the rates we observed (18.6 per 100,000). In contrast a study in rural western Kenya using relatively similar methods reported markedly higher rates of pneumococcal pneumonia among adults: 520 per 100,000 among human immunodeficiency virus (HIV)-uninfected, and 6710 per 100,000 among those infected with HIV [30], which is a strong risk factor for pneumococcal disease [31, 32]. Although an important difference is that the Kenyan study includes pneumococcal pneumonia outpatients (53%), the rates are still an order of magnitude higher than our study even after excluding outpatients. In addition to HIV, other risk factors for pneumococcal pneumonia, such as those associated with poverty [33], may explain some of the variability observed across regions. The rates of pneumococcal pneumonia that were observed in Guatemala are slightly higher than those reported by the Etiology of Pneumonia in the Community study in the US (12 per 100,000 adults per year) [22]. The vast difference in incidence estimates across sites highlights the need for more population-based studies of adult pneumococcal burden, particularly in resource-poor settings.

No clear seasonal pattern for pneumococcal disease was observed. In temperate regions, pneumococcal infection rates increase during the winter months and decline in the summer [31, 34], a pattern that is likely impacted by peaks in viral infections [35]. In Guatemala, RSV burden is greatest from July to November, and influenza A virus cases occur most frequently from March to July [14, 36, 37]; these non-overlapping peaks may make it harder to observe associations between increases in pneumococcal pneumonia and increases in viral infections. It has been described that respiratory viruses predispose to secondary bacterial infections, and there is evidence that influenza virus alters the host in a way that predisposes to adherence, invasion and induction of disease by pneumococcus [38–40]. Among adults with pneumococcal pneumonia in this study, 28% had at least one virus detected, and 11% had concurrent influenza infection, suggesting that achieving better control of influenza in adults in Guatemala could potentially reduce the burden of pneumococcal disease. Since 2007, influenza vaccination has been recommended for adults aged ≥60 years in Guatemala, although coverage has remained low.

PCV13 was introduced in the infant immunization program in Guatemala in November 2012 [41]. At this time, PAHO does not recommend the use of PCV for adults [42]. In the USA and in almost all European countries, policies to vaccinate elderly and ´at risk´ adults for the 23-valent pneumococcal polysaccharide vaccine (PPSV23) have been in place for more than two decades [43]. In August 2014, the Advisory Committee on Immunization Practices (ACIP)recommended that both PCV13 an PPSV23 be routinely administered in series to all adults ≥65 years [44]. Indirect protection against invasive pneumococcal disease [12, 45, 46] and pneumonia [11, 12, 47, 48] in adults has been reported from high-income settings following PCV introduction for infants. The development of herd effects in resource-poor settings may be affected by higher rates of pneumococcal carriage, the force of transmission of *S*. *pneumoniae* from children to adults (related to crowding, exposure to indoor air pollution, sanitation/hygiene), the robustness of the immune response in vaccinated children (which may be affected by chronic conditions such as malnutrition), and vaccine coverage. Herd protection can dramatically alter cost-effectiveness analyses of PCV [49, 50]; for a middle-income country such as Guatemala, the additional benefit of preventing adult pneumonia can have tremendous policy relevance. These data can be used as a baseline to provide that evidence.

The findings of this study are subject to several limitations. Blood cultures are not routinely used for ARI patients and only detect bacteremic cases that are not already on antibiotic treatment [8, 51, 52]. Urine antigen testing can detect non-bacteremic pneumococcal pneumonia in adults, yet with a suboptimal sensitivity [3, 9, 26, 27], so some cases will be missed. Use of newer, more sensitive urine antigen assays could provide a more accurate estimation of pneumococcal disease burden in adults [5, 6, 53, 54]. Our surveillance is hospital-based; the findings cannot be extrapolated to persons with ARI that seek care elsewhere or do not seek medical care. In estimating the incidence of hospitalized pneumococcal pneumonia, we attempted to adjust for lack of testing, non-enrollment and proportion of population that seeks care at surveillance hospitals; however the proportion seeking care at surveillance hospitals may have changed over time, which would affect the observed and adjusted incidence. This study only estimates the incidence of hospitalized pneumococcal pneumonia, and failure to account for non-hospitalized cases may have resulted in underestimation of the true burden of pneumococcal pneumonia in adults.

Despite the limitations, our findings provide insight into the burden of hospitalized pneumococcal pneumonia in adults. Information on burden of pneumonia is essential for evidence-based public health policies and can guide decisions about the use of prevention interventions such as vaccines. These data provide a baseline against which to measure the indirect effects of the recent introduction of PCV in Guatemala.

## Supporting Information

S1 Dataset Population. Number people living in the pneumonia surveillance catchment area by department and year.
(XLSX)

S2 Dataset Pneumococcal pneumonia patients. Clinical characteristics of individual patients with pneumococcal pneumonia.
(XLSX)

S3 Dataset ARI and pneumococcal pneumonia cases by year. Weekly numbers of ARI and pneumococcal pneumonia cases.
(XLSX)

S1 Table ARI and pneumococcal pneumonia by age group. Number of ARI and pneumococcal pneumonia cases by age group.
(XLSX)

S2 Table Factors for incidence rate adjustments. Factors used to adjust the incidences according to department, year and type of adjustment.
(XLSX)
